# Correction: Pulmonary embolism in a patient with eltrombopag-treated aplastic anaemia and paroxysmal nocturnal haemoglobinuria clone during COVID-19 pneumonia

**DOI:** 10.1186/s12959-022-00412-z

**Published:** 2022-09-13

**Authors:** Alessandro Bosi, Wilma Barcellini, Bruno Fattizo

**Affiliations:** 1 Haematology Unit, Fondazione IRCCS Ca’ Granda Ospedale Maggiore Policlinico, Via Francesco Sforza 35, 20100 Milan, Italy; 2 Department of Oncology and Haemato-oncology, University of Milan, Milan, Italy

**Correction: Thrombosis J 20, 46 (2022)**

**https://doi.org/10.1186/s12959-022-00407-w**

Following publication of the original article [1], it was reported that the Given and Family names of the authors were transposed. The correct authorship panel is given in this Correction article and the original article [1] has been updated.
